# An Integrative Comparative Study Between Digoxin and Amiodarone as an Emergency Treatment for Patients With Atrial Fibrillation With Evidence of Heart Failure: A Systematic Review and Meta-Analysis

**DOI:** 10.7759/cureus.26800

**Published:** 2022-07-13

**Authors:** Hany A Zaki, Khalid Bashir, Haris Iftikhar, Waleed Salem, Eslam Hussein Mohamed, Helmy M Elhag, Mohamed Hendy, Ahmed Abdu O Kassem, Emad El-Din Salem, Amr Elmoheen

**Affiliations:** 1 Emergency Medicine, Hamad Medical Corporation, Doha, QAT; 2 Medicine, Qatar University, Doha, QAT; 3 Accident and Emergency, Hamad Medical Corporation, Doha, QAT; 4 Anesthesia, Armed Forces Hospitals, Cairo, EGY

**Keywords:** systematic review and meta-analysis, pharmacological agents, chronic heart failure, anti-arrhythmic drugs, atrial fibrillation (af), digoxin, amiodarone

## Abstract

The emergency treatment of atrial fibrillation (AF) involves utilizing two strategies. The first strategy normally involves permitting the atrial fibrillation to persevere as the ventricular rate is controlled. The other method involves utilizing anti-arrhythmic drugs in cardioversion and attempting to maintain sinus rhythm. Different pharmacological treatments, including digoxin and amiodarone, have been used to manage AF. A literature review on amiodarone and digoxin in the treatment of AF among patients with heart failure (HF) has shown that both drugs have potential risks. Therefore, we are conducting this systematic review and meta-analysis to compare the effectiveness of amiodarone and digoxin in the treatment of AF among patients with evidence of HF.

A literature search of relevant articles was conducted on six electronic databases (PubMed, Web of Science, Medline, ScienceDirect, Cochrane Library, and Google Scholar) from 2000 to 2022. The search yielded seven studies that had met the inclusion criteria. Our meta-analysis of four studies showed that there was no significant difference in the reduction of heart rate after treatment with either amiodarone or digoxin (mean difference (MD): -5.44; 95% confidence interval (CI): -9.53 to -1.34; I^2^ = 25%; p = 0.26). On the other hand, the statistical analysis showed that amiodarone had a better effect on the conversion to sinus rhythm than digoxin (63% versus 35%, respectively).

Based on evidence from our meta-analysis, the clinical effect of amiodarone and digoxin in the emergency treatment of AF on heart rate control was unclear. However, amiodarone has a significant impact on the restoration of sinus rhythm compared with digoxin and can be considered the first-line drug regimen in conversion to sinus rhythm for AF patients with evidence of heart failure. However, the use of amiodarone and digoxin is complicated by adverse events and all-cause mortality.

## Introduction and background

Heart failure (HF) is a hazardous disease whose occurrence in the past decade has been growing at an alarming rate. As per the 2021 American Heart Association Statistical Update, the prevalence of HF was approximate six million, which is about 1.8% of the United States’ total population [1,2]. Estimates from other surveys have revealed that the prevalence of HF in the United States and Canada is assessed at 1.5%-1.9% of the population and 1%-2% in Europe [3]. This increasing trend in heart failure (CHF) is also associated with increased morbidity and mortality from progressive pump dysfunction and arrhythmias. One of the most common arrhythmias related to HF is atrial fibrillation (AF), which is prevalent in approximately 2% of the general population and about 3-6 million individuals in the United States [4]. AF is depicted as a supraventricular tachyarrhythmia typically characterized by the irregular, frequent, rapid ventricular response when the atrioventricular (AV) node conduction is intact. Previous studies have reported that atrial fibrillation is more frequent in men than in women. For instance, a study of over 38 years reported that AF was more common in men (2.2%) as opposed to women (1.7%) [5].

Additionally, AF has been depicted as the most widely recognized cardiac arrhythmia, representing about 35% of hospital admissions for cardiac arrhythmias [6], and is, therefore, the most commonly recognized cardiac arrhythmia treated in the emergency department. There has been growing evidence that AF can be managed/treated in the emergency department with practically no requirement for hospital admission [7,8]. In the emergency department, AF can be overseen utilizing two strategies. The first strategy normally involves permitting the atrial fibrillation to persevere as the ventricular rate is controlled. The other method consists of using anti-arrhythmic drugs in cardioversion and attempting to maintain sinus rhythm. The efficacy of rhythm and rate control in the treatment of AF in patients with HF was discussed in a recently published randomized clinical trial [9]. Furthermore, the outcomes of this trial revealed that rate and rhythm control showed no huge contrast in deaths from cardiovascular causes (27% in the rhythm-control group and 25% in the rate-control group; p=0.59). Similarly, there was no distinction in the secondary outcomes such as all-cause deaths, worsening HF, or stroke for rhythm and rate control. Additionally, the study’s outcome showed that rate control effectively eliminated the need for repeated cardioversion and reduced hospitalization rates. Other studies have shown no significant difference in the treatment of AF by rate and rhythm control strategies [10,11].

Several treatment options, either pharmacological or non-pharmacological, have been used in rate control for patients with AF and HF. Previous studies have reported that the use of Class IC anti-arrhythmic drugs in the treatment of AF among patients with HF is limited due to their pro-arrhythmic and adverse inotropic effects [12,13]. Other anti-arrhythmic drugs, such as amiodarone and dofetilide, are pharmacological agents of choice in maintaining sinus rhythm among AF patients. Despite amiodarone showing significant conversion to sinus rhythm, it is associated with increased risks of sudden cardiac death for patients with NHYA class III heart failure. Moreover, the extracardiac toxicity of amiodarone may also hinder the long-term use of the drug.

On the other hand, digoxin has been used as a rate control agent for AF management, especially for patients with HF. However, a previous study in which 38,898 patients were assigned to the digoxin group revealed that the drug was associated with increased mortality compared to other rate-controlling drugs [14]. Further clinical studies have also raised concerns about the safety of digoxin during AF management. A population-based study comparing AF patients treated with or without digoxin showed that in the absence of an anticoagulant, digoxin was associated with a 1.4-fold increase in the risk of ischemic stroke [15]. Similarly, a meta-analysis of digoxin mortality revealed that digoxin was associated with higher mortality [16].

The literature review conducted on amiodarone and digoxin in the treatment of AF among patients with HF shows that both drugs have potential risks. Therefore, we are running this systematic review and meta-analysis to compare the effectiveness of amiodarone and digoxin in the treatment of AF among patients with evidence of HF. We hypothesize that digoxin will significantly improve the heart rate compared to amiodarone, while amiodarone will be more effective in achieving conversion to sinus rhythm among AF patients.

## Review

Methods

Literature Search

Using the Preferred Reporting Items for Systematic Reviews and Meta-Analyses (PRISMA) guidelines, two reviewers thoroughly searched six electronic databases including PubMed, Web of Science, Medline, ScienceDirect, Cochrane Library, and Google Scholar. The Boolean operators “AND” and “OR” together with MeSH terms were used in the search strategy, which was as follows: (“Amiodarone” OR “anti-arrhythmic drugs”) AND (“Digoxin” OR “Pharmacological agents”) AND (“Treatment” OR “Management”) AND (“Atrial fibrillation”) AND (“Heart failure” OR “chronic heart failure”). The reviewers also scoured the reference lists of the identified articles and other systematic reviews for additional studies.

Eligibility Criteria

All studies were screened using the inclusion and exclusion criteria before being included in this systematic review and meta-analysis. The inclusion criteria were as follows: studies written and published in the English language (the reviewers made this provision to avoid the loss of scientific meanings, which comes with a direct translation of scientific terms), studies that included human subjects only, studies that compared amiodarone and digoxin in the treatment of atrial fibrillation for patients with evidence of heart failure, and studies evaluating more than 10 patients.

On the other hand, studies were excluded from this systematic review due to the following reasons: studies were published in languages other than English, studies evaluated animal subjects, studies compared amiodarone and digoxin in the treatment of atrial flutter alone, studies excluded patients with heart failure, and studies independently compared either amiodarone or digoxin with other pharmacological treatments. Abstracts, letters to the editor, and other systematic reviews and meta-analyses were also excluded from this review.

Quality Assessment

**Figure 1:**
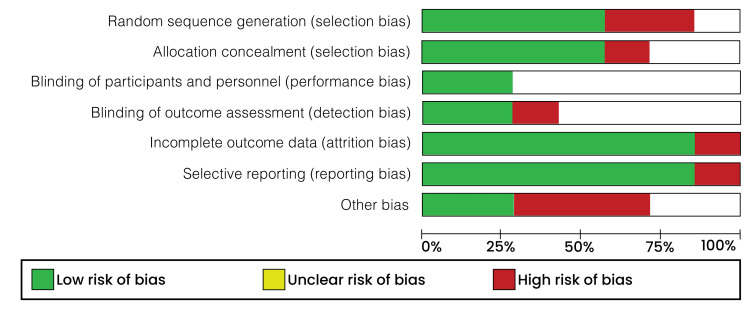
Risk of bias graph

**Figure 2:**
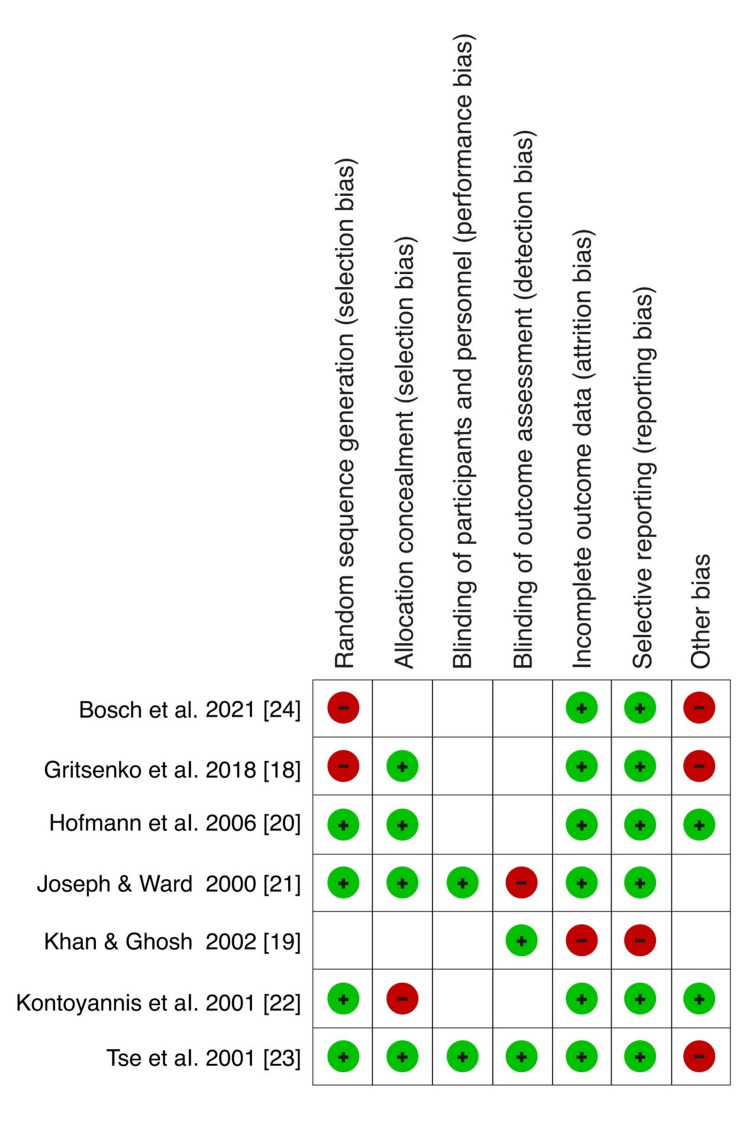
Risk of bias summary Gritsenko et al. (2018) [18], Khan and Ghosh (2002) [19], Hofmann et al. (2006) [20], Joseph and Ward (2000) [21], Kontoyannis et al. (2001) [22], Tse et al. (2001) [23], Bosch et al. (2021) [24]

The Cochrane Handbook for Systematic Reviews and Meta-Analyses was used in the quality assessment of every included randomized study [17]. The Cochrane risk of bias tool in the RevMan software (version 5.4.1) was used in the evaluation. During the assessment, the following elements were considered: selection, performance, attrition, and reporting bias. Each study element was then categorized as “low risk,” “high risk,” or “unclear risk.” Low risk of bias indicated that the element under consideration was sufficiently addressed, while a high risk of bias meant that the element under consideration was insufficiently addressed. On the other hand, unclear risk of bias meant that the element under study had very few details, and the reviewers’ judgment could not be made. The risk of bias graph is shown and illustrated in Figure 1, and the risk of bias summary is shown and illustrated in Figure 2.

Data Extraction

Two reviewers were tasked with retrieving and compiling relevant data from the included studies. The data retrieved included: author ID (author and year of publication), population characteristics, intervention, control intervention, follow-up period, and main outcomes. The patients’ characteristics included sample size, age, and sex. The dosages of amiodarone or digoxin were specified in the intervention and control sections. In case of disagreements in the data extraction process, the two reviewers consulted a third reviewer to help reconcile the debate. The primary outcomes of this systematic review were heart rate control and sinus rhythm conversion. On the other hand, adverse effects associated with either amiodarone or digoxin and all-cause mortality were used as secondary outcomes in this systematic review and meta-analysis.

Data Analysis

The RevMan software (version 5.4.1) was used in the meta-analysis of all outcomes. The effect size of continuous outcomes was calculated using mean difference (MD), while outcomes of discrete nature were assessed using odd’s difference. A random-effect model was used while using the eligibility criteria for the meta-analysis because it takes into consideration the study sample size and heterogeneity. A heterogeneity value of 50%-70% was considered moderate, while heterogeneity greater than 70% was considered substantial. A 95% confidence interval (CI) was also chosen because the number of studies included in this systematic review was small, limiting the statistical power. Forest plots with a significance level of 5% (p ≤ 0.05) were used in the meta-analysis of data.

Results

Search Results

**Figure 3:**
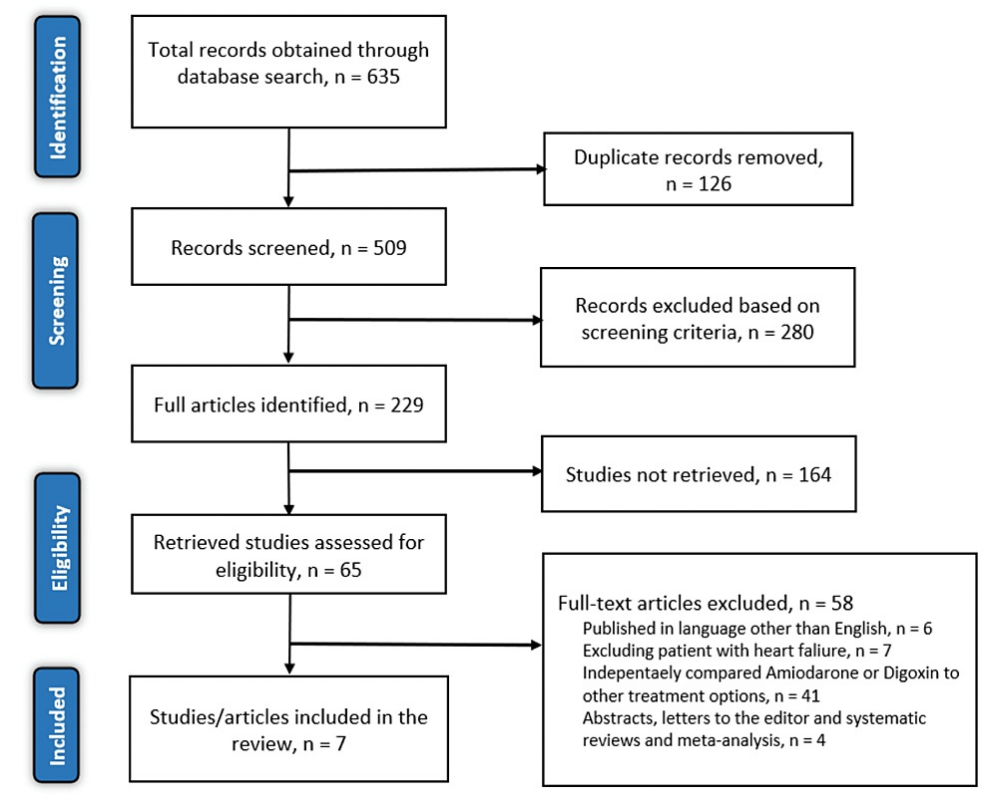
PRISMA flow diagram of the literature search results PRISMA: Preferred Reporting Items for Systematic Reviews and Meta-Analyses

An initial search through the six electronic databases mentioned earlier yielded 635 articles. The studies were then screened for duplicates, and 126 articles were excluded. Of the 509 remaining articles, 280 were excluded after screening the titles and abstracts, while 164 articles were not retrieved. The other articles were then screened using the eligibility criteria outlined earlier. Of the 65 articles assessed using the eligibility criteria, 58 were excluded due to the following reasons: six were published in other languages, seven evaluated atrial flutter only, 41 independently compared amiodarone or digoxin to other treatment measures, and eight were either abstracts, systematic reviews, and meta-analyses, or letters to the editor (Figure 3). Table 1 shows the study’s characteristics.

**Table 1:** Study characteristics AF: atrial fibrillation, LVEF: left ventricular ejection fraction, bpm: beats per minute

Author ID	Population	Intervention	Control	Follow-up period (hours)	Main outcomes
Gritsenko et al. (2018) [18]	268 patients (aged ≥18 years) with atrial fibrillation (AF)	32 patients (22 male and 10 female) were assigned to the amiodarone group	54 patients (38 male and 16 female) were assigned to the digoxin group	24	Rate control was accomplished following 24 hours in 29 (90.60%) patients in the amiodarone group and 52 (96.30%) patients in the digoxin bunch. Restoration to sinus rhythm was seen in a large portion of the population in the amiodarone group than in the digoxin group (50% versus 16.60%, respectively). Heart rates after 24 hours of treatment showed an inconsequential distinction in the two groups.
Khan and Ghosh (2002) [19]	153 patients (56 male and 97 females, with a mean age of 85.2 years) with AF	23 patients with new-onset AF were treated using digoxin	10 patients with new-onset AF were treated using amiodarone	76	Of the 23 patients in the digoxin group, three returned to sinus rhythm, while two in the amiodarone group returned to sinus rhythm. Ten AF patients treated with digoxin died, while one patient in the amiodarone group died. Of the 10 patients treated with a combination of digoxin and amiodarone, nine reverted to sinus rhythm, while the one patient who failed to cardiovert died.
Hofmann et al. (2006) [20]	100 patients with AF	50 patients (28 male and 22 female) with a mean age of 68.3 + 13 years were dispensed to the amiodarone group and received 450 mg of amiodarone intravenous, followed by a flush of 10 mL saline solution	50 patients (28 male and 22 female) with a mean age of 69.3 + 13 years were allocated to the digoxin group, and 0.6 mg intravenous digoxin was administered to the patients	24	Conversion to normal sinus rhythm was seen in 14 patients in the amiodarone cohort and three patients in the digoxin cohort following 30 minutes of drug administration. After 60 minutes, sinus rhythm conversion was higher in the amiodarone group compared with the digoxin group (21 versus nine patients, respectively.) After 60 minutes of drug administration, the mean ventricular heart rates were 94.2 + 22 bpm and 105.3 + 22 bpm in the amiodarone and digoxin groups.
Joseph and Ward (2000) [21]	120 patients with new-onset AF	36 patients (20 male and 16 female, with a mean age of 64.9 + 2.0 years) in the digoxin group were subjected to 500 ug intravenous for over 30 minutes, followed by 250 ug oral dosage every six hours for four doses	39 patients in the amiodarone group were subjected to 5 mg/kg intravenously for over 30 minutes and then 400 mg orally every eight hours for six doses; 40 patients in the sotalol group were subjected to 1.5 mg/kg intravenously for over 30 minutes and then 80 mg orally every eight hours for six doses	48	At 24 hours, adequate rate control was observed in four of seven patients in the sotalol group, three of 12, and five of 18 in the amiodarone and digoxin groups, respectively. Cardioversion to sinus rhythm at 48 hours of drug administration was 28 (78%), 37 (94%), and 38 (95%) in the digoxin, amiodarone, and sotalol groups, respectively. Eight, three, and two patients in the digoxin, amiodarone, and sotalol groups witnessed adverse events associated with the drug regimens.
Kontoyannis et al. (2001) [22]	70 patients with acute myocardial infarction complicated with AF	26 patients in the digoxin group were subjected to intravenous 0.5 mg digoxin diluted in 10 mL of 5% glucose solution administered over five minutes; for AF persisting after one hour, 0.25 mg of digoxin was administered intravenously	16 patients in the amiodarone group were subjected to 300 mg of amiodarone hydrochloride diluted in 500 mL of 5% glucose solution administered over two hours, followed by a continuous infusion of 44 mg/hour until the restoration of sinus rhythm or up to 60 hours	96	Four of 16 and nine of 26 patients in the digoxin and amiodarone groups had a conversion to sinus rhythm after two hours. After 8- 96 hours, eight and nine patients in the digoxin and amiodarone groups had cardioversion to sinus rhythm. The sinus heart rate at the time of cardioversion was 91 ± 18 bpm, 81 ± 17 bpm, and 87 ± 18 bpm in the three respective groups.
Tse et al. (2001) [23]	16 patients (13 male and three females, with a mean age of 63 + 9 years) with chronic AF	Nine patients in the amiodarone group were subjected to 600 mg daily for one week as a loading dose and then 100 mg daily for 24 weeks	Seven patients in the digoxin group were subjected to 0.25 mg daily or 0.125 mg daily if bodyweight < 50 kg or serum creatinine > 200 mmol/L	24 weeks	No significant difference was observed in the percentage reduction of ventricular rate after 24 weeks in the two groups (27% + 13% versus 25% + 12%, for digoxin and amiodarone, respectively). A significant increase in left ventricular ejection fraction (LVEF) was observed in the digoxin compared with the baseline (0.71 + 0.13 versus 0.63 + 0.11, respectively), while no difference was noted in the amiodarone group (0.69 + 0.09 versus 0.66 + 0.11, respectively). At 24 weeks, no significant difference was observed in the quality of life and AF symptoms after treatment with either amiodarone or digoxin.
Bosch et al. (2021) [24]	666 patients (362 female and 304 males, with a mean age of 72 (12) years) with sepsis-associated atrial fibrillation	337 patients were assigned to the amiodarone group	67, 225, and 37 patients were enrolled in the beta-blocker, calcium channel blocker, and digoxin group	6	Six hours after medication therapy, the heart rate was significantly lowered in patients who received beta-blockers (110 bpm) compared with those who received digoxin (118 bpm); however, no difference was observed when compared with amiodarone (110 bpm). A high hospital mortality rate (39.20%) was observed in the amiodarone group compared with the digoxin group (13.50%).

Primary Outcomes

**Figure 4:**
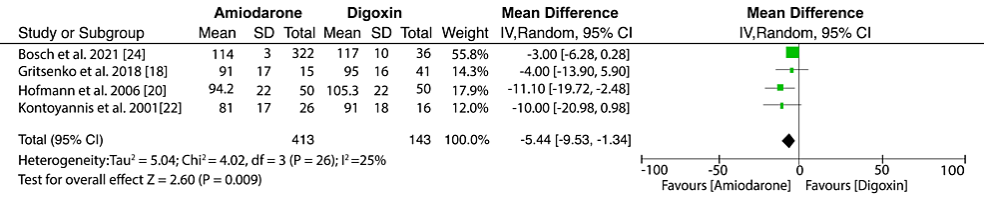
Forest plot of studies evaluating the effect of amiodarone versus digoxin in heart rate control Bosch et al. (2021) [24], Gritsenko et al. (2018) [18], Hofmann et al. (2006) [20], Kontoyannis et al. (2021) [22]

Meta-analysis of heart rates from four studies shows that amiodarone had a better effect than digoxin. However, the reduction in heart rates for AF patients showed no statistically significant difference (MD: -5.44; 95% CI: -9.53 to -1.34; I^2^ = 25%; p = 0.26) (Figure 4). Low heterogeneity (I^2^ = 25%) was recorded among studies that evaluated the effect of amiodarone versus digoxin in heart rate control. Consequently, there was a low variation of study outcomes from these studies.

Statistical analysis of the number of patients with successful rate control showed no statistical difference after treatment with either amiodarone or digoxin (Table 2).

**Table 2:** Analysis of the number of patients with improved rate control

Author ID	Amiodarone		Digoxin	
	Successful number of rate control	Total population	Successful number of rate control	Total population
Joseph and Ward [21]	30	39	21	36
Gritsenko et al. [18]	24	32	42	54
Tse et al. [23]	2	9	3	7
Total	56	80	66	97
Percentage	70%		68%	

On the other hand, statistical analysis of conversion to sinus rhythm shows that amiodarone had a significant impact compared to digoxin (Table 3).

**Table 3:** Amiodarone versus digoxin in sinus rhythm at the final follow-up period

Author ID	Amiodarone		Digoxin	
	Successful conversion to sinus rhythm	Total population	Successful conversion to sinus rhythm	Total population
Joseph and Ward [21]	37	39	28	36
Kontoyannis et al. [22]	16	16	18	26
Hofmann et al. [20]	21	50	9	50
Gritsenko et al. [18]	16	32	9	54
Khan and Ghosh [19]	2	10	3	23
Total	92	147	67	189
Percentage	63%		35%	

Secondary Outcomes

**Figure 5:**
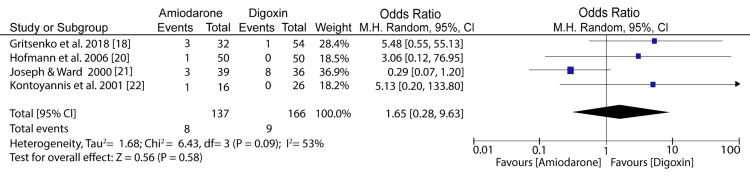
Forest plot of studies on adverse events related to amiodarone and digoxin Gritsenko et al. (2018) [18], Hofmann et al. (2006) [20], Joseph and Ward (2000) [21], Kontoyannis et al. (2001) [22]

When the effect of digoxin was compared to amiodarone on adverse events, the meta-analysis showed no significant difference between the two groups and slightly significant heterogeneity (odds ratio (OR): 1.65; 95% CI: 0.28 to 9.63; I^2^ = 53%; p = 0.09) (Figure 5).

**Figure 6:**
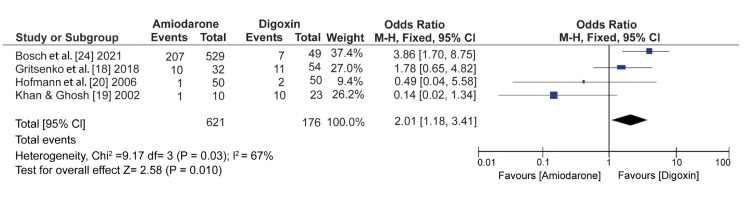
Forest plot of studies comparing amiodarone to digoxin in all-cause mortality Bosch et al. (2021) [24], Gritsenko et al. (2018) [18], Hofmann et al. (2006) [20], Khan and Ghosh (2002) [19]

On the other hand, digoxin showed a significant decrease in all-cause mortality compared with amiodarone and significant (OR: 2.01; 95% CI: 1.18 to 3.41; I^2^ = 67%; p = 0.03) (Figure 6).

Discussion

This systematic review and meta-analysis were designed to compare the effectiveness of amiodarone and digoxin in the treatment of AF among patients with evidence of heart failure. Despite amiodarone showing a better effect on reducing heart rate, our analysis showed that the difference did not reach a statistical significance (p = 0.26). Similarly, when comparing amiodarone with digoxin on the number of adverse events, there was no significant difference between the two groups. However, a meta-analysis of all-cause mortality showed that digoxin was more effective than amiodarone.

This systematic review hypothesized that digoxin would have a better effect on reducing heart rate among patients with AF. However, the results of our meta-analysis showed no significant difference for AF patients treated with either amiodarone or digoxin These results are supported by a previous meta-analysis that showed that digoxin was inferior to amiodarone in controlling heart rates in patients with new-onset AF within six hours of treatment. Despite digoxin being inferior, the study reports that the difference was not significant (MD: 14.7 bpm; trial sequential analysis (TSA)-adjusted CI: -0.58 to 30.0; I^2^ = 42%; p < 0.00001) [25]. Our statistical analysis has also shown that 70% and 68% of people treated with amiodarone and digoxin, respectively, had achieved rate control after the treatment measures. These results indicate no statistically significant difference between the two treatment options. However, some of the included studies have provided some contradicting results. Gritsenko et al. [18] reported that after 24 hours, more patients in the digoxin group had achieved rate control compared with patients in the amiodarone group. In previous studies where AF patients showed no evidence of heart failure, digoxin has also shown some contradicting results on the ventricular rate. Siu et al. [26] evaluated the effectiveness between diltiazem, digoxin, and amiodarone among patients who presented to the emergency department with acute-onset symptomatic AF. The results of this trial showed that of 150 patients, 119 patients had achieved ventricular rate control within the first 24 hours. Of these patients who achieved rate control, patients randomized to the digoxin and amiodarone groups showed no difference (74% versus 74%); however, patients in the diltiazem group showed a significantly higher rate control (90%). Similarly, a 2004 randomized trial reported that digoxin showed a significantly lower rate of control compared with sotalol and amiodarone [27]. The results of this study showed that most patients treated with digoxin did not achieve a heart rate of less than 100 bpm until six hours. On the other hand, sotalol and amiodarone showed a rapid rate control, of which most of the patients achieved a heart rate of less than 100 bpm within 30 minutes.

We also hypothesized that amiodarone would significantly convert the AF to sinus rhythm compared with digoxin. The results of the statistical analysis have supported this hypothesis by showing that 63% of patients treated with amiodarone had converted to standard sinus reason while only 35% of the patients had achieved sinus rhythm. These results are supported by a recent meta-analysis of three trials that showed that amiodarone was an effective treatment in converting AF to sinus rhythm compared with digoxin [25]. Similarly, a meta-analysis that compared amiodarone to other pharmacological treatments, including digoxin, showed that amiodarone was more efficient in conversion to sinus rhythm in AF patients [28]. Other randomized trials have also demonstrated that amiodarone is an effective anti-arrhythmic drug in conversion to sinus rhythm. Hou et al. [29] evaluated the treatment of recent-onset atrial fibrillation using amiodarone and digoxin and found that 24 of 26 cases of AF had converted back to sinus rhythm 24 hours after the administration of amiodarone, while only 17 of 24 cases of AF in the digoxin group had converted to sinus rhythm. Despite this high rate of conversion observed after digoxin administration, it was recorded that of the 17 episodes of conversion to sinus rhythm, four patients had a recurrence of AF, while only one of the patients in the amiodarone group showed recurrence of AF. Similarly, a 2000 randomized study evaluating the effectiveness of diltiazem, amiodarone, and digoxin among patients with persistent AF showed that 85 of 115 patients had achieved restoration to sinus rhythm [30]. The results of this study showed that 30 of 33 AF patients in the amiodarone group had restored sinus rhythm, while only 19 of 29 AF patients in the digoxin group had converted to sinus rhythm. Twenty-four hours after the sinus rhythm restoration, three (12%) of the patients in the digoxin group had a recurrence of AF, while only one (3%) patient in the amiodarone group had a recurrence of AF. A one-month follow-up showed that a lower AF recurrence rate was observed in the amiodarone group compared to the diltiazem and digoxin groups (8 (28%) versus 17 (56%) versus 12 (78%), respectively). Evidence has also shown that amiodarone has no significant difference in the conversion to sinus rhythm compared to other treatment measures. Galve et al. [31] reported that AF patients treated with 5 mg/kg intravenous amiodarone for 30 minutes followed by 1,200 mg for over 24 hours had no significant difference in conversion rate compared to the placebo. Similarly, Bianconi et al. [32] reported no difference in conversion to sinus rhythm for patients treated with a 15-minute infusion of amiodarone compared with a placebo. A study comparing amiodarone to flecainide and propafenone showed that amiodarone given at 5 mg/kg for 20 minutes had a significantly lower conversion rate compared to the other drug regimens [33].

Despite the effectiveness of amiodarone in converting AF to sinus rhythm, the meta-analysis of four studies has shown that it is associated more with all-cause mortality than digoxin. However, the studies included in this review show contradicting results. Hofmann et al. [20] reported that of 100 patients equally randomized to the amiodarone and digoxin group, three deaths occurred; one of the three patients randomized into the amiodarone group died eight days after drug administration, while two patients in the digoxin died due to recurrent pulmonary embolism and developed pulmonary edema. However, this study reports that none of the patients had died directly from the drugs. Similarly, a 2002 study evaluating the effectiveness of pharmacological agents on AF showed that 14 of 53 patients with new-onset AF had died [19]. The results of this study show that 10 of 23 and one of 10 patients in the digoxin and amiodarone groups, respectively, had died. Previous studies comparing amiodarone to other drug treatments have also shown that despite amiodarone restoring sinus rhythm, it is limited by mortality rates. Singh et al. [34] evaluated the effectiveness of amiodarone and sotalol in the treatment of AF and found that 258 (27.1%) patients in the amiodarone group had a spontaneous conversion to sinus rhythm, while 244 (24.2%) patients in the sotalol group had achieved sinus rhythm. Despite amiodarone showing a significant conversion, the study reports that its use was affected by the number of deaths observed. The results show that 13 deaths (six sudden) were observed in the amiodarone group. A large cohort study of 122,465 patients also reported that amiodarone use for the treatment of AF was associated with an increased risk of death [35]. Additionally, the use of amiodarone in treating patients with an increased risk of sudden cardiac deaths, such as those with heart failure, has also been associated with high all-cause mortality rates. Our recent meta-analysis comparing the effectiveness of implantable cardioverter-defibrillator (ICD) to amiodarone in decreasing mortality from sudden cardiac deaths showed that amiodarone was highly associated with all-cause mortality compared with ICD (OR: 1.36; 95% CI: 1.06 to 1.74; I^2^ = 57%; p = 0.03) [36].

Evidence has also shown that adverse events limit the effectiveness of digoxin and amiodarone in treating AF. Our meta-analysis of three studies has demonstrated that amiodarone and digoxin showed no significant difference in the number of patients experiencing adverse events after treatment using the drug regimens. The reported adverse events associated with either amiodarone or digoxin vary in different studies. Joseph and Ward [21] reported that eight and three patients in the digoxin and amiodarone groups, respectively, experienced adverse events. The study shows that two events of bradycardia were observed in the two groups, of which each group accounted for one event. Additionally, six and two patients in the digoxin and amiodarone groups, respectively, experienced left ventricular failure, while only one event of stroke was observed among patients randomized to the digoxin group. Kontoyannis et al. [22] reported that only one case of adverse event was observed between the amiodarone and digoxin groups. It was reported that despite restoration to sinus rhythm, one patient in the amiodarone group developed first degree of atrioventricular (AV) block. Hofmann et al. [20] also reported that 24 hours after amiodarone administration, one case of superficial phlebitis was observed. However, the study reported that the event was improved by treatment using local topic therapy. A follow-up of this case revealed that the amiodarone therapy had not been followed with the usual 10 mL saline flush as required by the drug protocol. In addition, in the study by Gritsenko et al. [18], there were four cases of adverse events in the amiodarone and digoxin groups. Of the four cases, one case of bradycardia was observed in the amiodarone group, while two cases and one case of hypotension were observed in the amiodarone and digoxin groups, respectively. Similarly, a previous study reported that one patient with severe heart failure and AF treated with amiodarone was reported to have an aggravation of HF one hour after drug administration [29]. The patient had to be discontinued from the amiodarone administration and was treated using diuretics and positive inotropic until stabilization. Another patient in this study was recorded to have severe bradycardia followed by cardiac arrest two hours after amiodarone administration. It is reported that the patient died despite aggressive attempts at resuscitation. Phlebitis was also observed in one patient who was treated with amiodarone.

Evidence has also shown that combining digoxin and amiodarone has a significant effect on the treatment of AF for patients with evidence of heart failure. Kontoyannis et al. [22] reported that 28 patients randomized to the digoxin plus amiodarone (D+AM) group had a significant conversion to sinus rhythm after two hours of therapy compared with patients randomized to digoxin and amiodarone groups. The results of conversion to sinus rhythm at 96 hours revealed that all patients in the D+AM had attained sinus rhythm, while eight patients in the digoxin group had not achieved sinus rhythm. Similarly, Khan and Ghosh [19] reported that of 10 patients with new-onset AF randomized to the combination group, nine had achieved sinus rhythm. However, previous studies have shown that a combination of amiodarone and digoxin is associated with high all-cause mortality compared to digoxin alone. A 2020 study evaluating 4,133 AF patients showed that significantly higher all-cause mortality rates were observed in the combination group than in the digoxin group (37.3% versus 26.9%, respectively) [37]. However, the study shows no significant difference in sudden cardiac groups in the two groups (6.7% versus 5.9% for digoxin and combination groups, respectively).

Limitations

The primary limitation of this systematic review and meta-analysis is the moderately high heterogeneity observed in the analysis of adverse events (53%) and all-cause mortality (67%). This high heterogeneity was expected because some studies included in this systematic review had variably large populations compared to others. This high heterogeneity should be considered when interpreting the results of our meta-analysis. Similarly, the use of heart rate control as a primary outcome may be questionable. This is because heart rate control has been established as a primary outcome in reducing the risk of cardiovascular events and deaths, but its clinical relevance in the treatment of AF has not been fully established [38,39]. Additionally, our eligibility criteria only allowed the inclusion of studies published in English only. Excluding studies published in other languages may have omitted some relevant outcomes that would have improved our meta-analysis.

## Conclusions

Based on evidence from our meta-analysis, the clinical effect of amiodarone and digoxin in the emergency treatment of AF on heart rate control was unclear. The results showed that amiodarone had a better effect in reducing heart rate; however, the difference with digoxin administration was insignificant. Our systematic review and meta-analysis have also shown that amiodarone significantly impacts the restoration of sinus rhythm compared with digoxin. These results indicate that amiodarone can be used as the first-line drug regimen in conversion to sinus rhythm for AF patients with evidence of heart failure. However, the long-term results of digoxin and amiodarone are unclear because no trial in this systematic review reported the long-term effects of the drug regimens in the treatment of AF. Therefore, there is a need for more clinical trials to be conducted to help assess the clinical impact of either amiodarone or digoxin in the treatment of AF. Our meta-analysis has shown that despite amiodarone being effective in conversion to sinus rhythm, it is associated with an increased risk of all-cause mortality and should be used comparably.

## References

[1] Roger VL (2021). Epidemiology of heart failure: a contemporary perspective. Circ Res.

[2] Virani SS, Alonso A, Aparicio HJ (2021). Heart disease and stroke statistics-2021 update: a report from the American Heart Association. Circulation.

[3] Ponikowski P, Anker SD, AlHabib KF (2014). Heart failure: preventing disease and death worldwide. ESC Heart Fail.

[4] Kornej J, Börschel CS, Benjamin EJ, Schnabel RB (2020). Epidemiology of atrial fibrillation in the 21st century: novel methods and new insights. Circ Res.

[5] Kannel WB, Wolf PA, Benjamin EJ, Levy D (1998). Prevalence, incidence, prognosis, and predisposing conditions for atrial fibrillation: population-based estimates. Am J Cardiol.

[6] Wakai A, O'Neill JO (2003). Emergency management of atrial fibrillation. Postgrad Med J.

[7] Connors S, Dorian P (1997). Management of supraventricular tachycardia in the emergency department. Can J Cardiol.

[8] Li H, Easley A, Barrington W, Windle J (1998). Evaluation and management of atrial fibrillation in the emergency department. Emerg Med Clin North Am.

[9] Roy D, Talajic M, Nattel S (2008). Rhythm control versus rate control for atrial fibrillation and heart failure. N Engl J Med.

[10] Van Gelder IC, Hagens VE, Bosker HA (2002). A comparison of rate control and rhythm control in patients with recurrent persistent atrial fibrillation. N Engl J Med.

[11] Carlsson J, Miketic S, Windeler J (2003). Randomized trial of rate-control versus rhythm-control in persistent atrial fibrillation: the Strategies of Treatment of Atrial Fibrillation (STAF) study. J Am Coll Cardiol.

[12] Naccarelli GV, Wolbrette DL, Khan M, Bhatta L, Hynes J, Samii S, Luck J (2003). Old and new anti-arrhythmic drugs for converting and maintaining sinus rhythm in atrial fibrillation: comparative efficacy and results of trials. Am J Cardiol.

[13] Matalka MS, Deedwania PC (2001). Atrial fibrillation in patients with heart failure: pharmacologic options. Congest Heart Fail.

[14] Chao TF, Liu CJ, Tuan TC (2015). Rate-control treatment and mortality in atrial fibrillation. Circulation.

[15] Chao TF, Liu CJ, Chen SJ (2014). Does digoxin increase the risk of ischemic stroke and mortality in atrial fibrillation? A nationwide population-based cohort study. Can J Cardiol.

[16] Ziff OJ, Lane DA, Samra M (2015). Safety and efficacy of digoxin: systematic review and meta-analysis of observational and controlled trial data. BMJ.

[17] (2019). Cochrane handbook for systematic reviews of interventions, second edition.

[18] Gritsenko D, Paris D, Aitken SL, Lee YI, Altshuler J (2018). Amiodarone versus digoxin for rate control in critically ill patients with rapid atrial fibrillation or flutter. J Emerg Crit Care Med.

[19] Khan SA, Ghosh P (2002). Management of atrial fibrillation in older patients. J Pak Med Assoc.

[20] Hofmann R, Steinwender C, Kammler J, Kypta A, Leisch F (2006). Effects of a high dose intravenous bolus amiodarone in patients with atrial fibrillation and a rapid ventricular rate. Int J Cardiol.

[21] Joseph AP, Ward MR (2000). A prospective, randomized controlled trial comparing the efficacy and safety of sotalol, amiodarone, and digoxin for the reversion of new-onset atrial fibrillation. Ann Emerg Med.

[22] Kontoyannis DA, Anastasiou-Nana MI, Kontoyannis SA, Zaga AK, Nanas JN (2001). Intravenous amiodarone decreases the duration of atrial fibrillation associated with acute myocardial infarction. Cardiovasc Drugs Ther.

[23] Tse HF, Lam YM, Lau CP, Cheung BM, Kumana CR (2001). Comparison of digoxin versus low-dose amiodarone for ventricular rate control in patients with chronic atrial fibrillation. Clin Exp Pharmacol Physiol.

[24] Bosch NA, Rucci JM, Massaro JM (2021). Comparative effectiveness of heart rate control medications for the treatment of sepsis-associated atrial fibrillation. Chest.

[25] Sethi NJ, Nielsen EE, Safi S, Feinberg J, Gluud C, Jakobsen JC (2018). Digoxin for atrial fibrillation and atrial flutter: a systematic review with meta-analysis and trial sequential analysis of randomised clinical trials. PLoS One.

[26] Siu CW, Lau CP, Lee WL, Lam KF, Tse HF (2009). Intravenous diltiazem is superior to intravenous amiodarone or digoxin for achieving ventricular rate control in patients with acute uncomplicated atrial fibrillation. Crit Care Med.

[27] Thomas SP, Guy D, Wallace E (2004). Rapid loading of sotalol or amiodarone for management of recent onset symptomatic atrial fibrillation: a randomized, digoxin-controlled trial. Am Heart J.

[28] Letelier LM, Udol K, Ena J, Weaver B, Guyatt GH (2003). Effectiveness of amiodarone for conversion of atrial fibrillation to sinus rhythm: a meta-analysis. Arch Intern Med.

[29] Hou ZY, Chang MS, Chen CY, Tu MS, Lin SL, Chiang HT, Woosley RL (1995). Acute treatment of recent-onset atrial fibrillation and flutter with a tailored dosing regimen of intravenous amiodarone. A randomized, digoxin-controlled study. Eur Heart J.

[30] Villani GQ, Piepoli MF, Terracciano C, Capucci A (2000). Effects of diltiazem pretreatment on direct-current cardioversion in patients with persistent atrial fibrillation: a single-blind, randomized, controlled study. Am Heart J.

[31] Galve E, Rius T, Ballester R, Artaza MA, Arnau JM, García-Dorado D, Soler-Soler J (1996). Intravenous amiodarone in treatment of recent-onset atrial fibrillation: results of a randomized, controlled study. J Am Coll Cardiol.

[32] Bianconi L, Castro A, Dinelli M, Alboni P, Pappalardo A, Richiardi E, Santini M (2000). Comparison of intravenously administered dofetilide versus amiodarone in the acute termination of atrial fibrillation and flutter. A multicentre, randomized, double-blind, placebo-controlled study. Eur Heart J.

[33] Martínez-Marcos FJ, García-Garmendia JL, Ortega-Carpio A, Fernández-Gómez JM, Santos JM, Camacho C (2000). Comparison of intravenous flecainide, propafenone, and amiodarone for conversion of acute atrial fibrillation to sinus rhythm. Am J Cardiol.

[34] Singh BN, Singh SN, Reda DJ (2005). Amiodarone versus sotalol for atrial fibrillation. N Engl J Med.

[35] Ullal AJ, Than CT, Fan J (2015). Amiodarone and risk of death in contemporary patients with atrial fibrillation: findings from The Retrospective Evaluation and Assessment of Therapies in AF study. Am Heart J.

[36] Zaki HA, Shaban E, Bashir K, Iftikhar H, Zahran A, Salem W, Elmoheen A (2022). A comparative study between amiodarone and implantable cardioverter-defibrillator in decreasing mortality from sudden cardiac death in high-risk patients: a systematic review and meta-analysis. Cureus.

[37] Chiang JY, Chen PC, Yang YH (2020). Digoxin-amiodarone Combination is associated with excess all-cause mortality in patients with atrial fibrillation. Sci Rep.

[38] Wyse DG (2004). Overview of endpoints in atrial fibrillation studies. Heart Rhythm.

[39] Böhm M, Reil JC (2013). Heart rate: surrogate or target in the management of heart failure?. Heart.

